# Metabolic and Transcriptomic Analyses Reveal Different Metabolite Biosynthesis Profiles between Purple and Green Pak Choi

**DOI:** 10.3390/ijms241813781

**Published:** 2023-09-07

**Authors:** Jinglei Wang, Tianhua Hu, Yidi Wang, Wuhong Wang, Haijiao Hu, Qingzhen Wei, Yaqin Yan, Chonglai Bao

**Affiliations:** 1 Institute of Vegetables Research, Zhejiang Academy of Agricultural Sciences, Hangzhou 310021, China; syauwjl@163.com (J.W.); hutianh@126.com (T.H.); 18858794853@163.com (Y.W.); hongge5@163.com (W.W.); huhj0571@126.com (H.H.); weiqz@mail.zaas.ac.cn (Q.W.); zkyyanyaqin@163.com (Y.Y.); 2 College of Horticulture Science, Zhejiang A&F University, Hangzhou 311300, China

**Keywords:** pak choi, transcriptomes, metabolism, anthocyanin, *MYB2*

## Abstract

Pak choi is one of the most important leafy vegetables planted in East Asia and provides essential nutrients for the human body. Purple pak choi differs mainly in leaf colour but exhibits distinct nutritional profiles from green pak choi. In this study, we performed metabolic and transcriptomic analyses to uncover the mechanisms underlying the differences in metabolite biosynthesis profiles between the two pak choi varieties. Metabolite profiling revealed significant differences in the levels of metabolites, mainly amino acids and their derivatives and flavonoids. Furthermore, 34 flavonoids significantly differed between green and purple pak choi leaves, and cyanidin and its derivative anthocyanins were abundant in purple pak choi. In addition, we found that the structural genes *CHS*, *DFR*, *ANS*, and *UGT75C1*, as well as the transcription factor *MYB2*, play a major role in anthocyanin synthesis. These results provide insight into the molecular mechanisms underlying leaf pigmentation in pak choi and offer a platform for assessing related varieties.

## 1. Introduction

Pak choi (*Brassica rapa* ssp. *chinensis*) is a vital leafy vegetable that has primarily been cultivated and consumed in China, Japan, and Korea for thousands of years. It belongs to the Brassica vegetable family [1]. In addition to its culinary uses, pak choi has been known to provide a variety of health benefits. It is low in calories and high in fibre, which can help support a healthy digestive system. It is also rich in vitamins A, C, and K, as well as potassium and calcium, which can help support overall health and well-being [2]. Hence, conducting research on the nutritional aspects of pak choi has the potential to be significant in addressing global concerns regarding public health and food security.

A variety of metabolic components have been detected in pak choi leaves, including organic acids, amino acids, and flavonoids [3]. Their relationship with morphology has been extensively studied, especially in purple pak choi [4]. The purple colour in the purple varieties of pak choi is mainly caused by anthocyanins which provide numerous benefits to both plants and humans. Fifteen types of anthocyanins have been identified in purple pak choi using high-performance liquid chromatography—electrospray ionization–mass spectrometry (HPLC—ESI—MS/MS) [5]. Anthocyanins have significant functions in plants, including a reduction in damage caused by cold and drought; UV irradiation; protection against viral, bacterial, and fungal infections; and the attraction of insects and animals for pollination and seed dispersal [6,7,8,9]. Additionally, these compounds have been recognized as food materials with physiological benefits due to their diverse biological activities, such as antioxidant and antiaging properties, as well as their potential to lower the risk of cancer and diabetes [10,11,12,13].

Anthocyanin biosynthesis is a significant secondary metabolic pathway in plants that utilizes phenylalanine as its primary precursor and is regulated by a series of enzymatic reactions. It has been extensively studied in various plant species, such as Arabidopsis, ornamental kale, and other plants [14,15,16]. Anthocyanins are flavonoid compounds that are synthesized from phenylalanine through a variety of enzymatic reactions to form colourless anthocyanidins. These anthocyanidins can then be modified through hydroxylation, acylation, glycosylation, or other substitution reactions to produce different anthocyanins. First, phenylalanine ammonialyase (PAL) drives the transformation of phenylalanine into cinnamic acid. Afterwards, a series of enzymatic reactions occur to generate dihydrokaempferol. These reactions involve enzymes such as chalcone synthase (CHS), chalcone isomerase (CHI), flavanone 3-hydroxylase (F3H), and flavonoid 3′-hydroxylase (F3′H). Finally, dihydroflavonol 4-reductase (DFR) converts dihydrokaempferol into unmodified and colourless anthocyanins, and anthocyanidin synthase (ANS) catalyses it into coloured anthocyanins [17,18]. Furthermore, the temporal and spatial control of anthocyanin accumulation is mediated by transcription factors (TFs) which regulate the expression of structural genes. The activity of these TFs is influenced by one or more TFs, primarily v-Myb avian myeloblastosis viral oncogene homologue (MYB), basic helix–loop–helix (bHLH), and WD repeat (WD40) families. Alternatively, the formation of a ternary TF complex (MYB-bHLH-WD40 or MBW) can also influence their activity. The mechanism of purple colouration in purple non-heading Chinese cabbage was investigated using metabolomic and transcriptomic approaches in previous reports [19,20], which used Caitai as the material. Hence, thorough research on metabolites and the transcriptome of purple pak choi leaves is lacking.

The leaves of pak choi serve as the primary organ responsible for nutrient storage and reproduction. Typically, the most prevalent cultivars of pak choi are those with green flesh. However, pak choi germplasm displays a wide range of colours. Purple pak choi typically contains higher levels of anthocyanins, making it a more valuable germplasm in breeding programs when compared to green pak choi. Nevertheless, it is still unclear whether pak choi of different colours possess similar nutritional properties and the related regulatory mechanisms at the transcriptional level. To address this issue, we employed a metabolome–transcriptome approach in our study to determine the metabolic and transcriptomic profiles of purple and green cultivars. The aim was to comprehensively identify natural variations in metabolites within pak choi and their corresponding regulatory mechanisms at the transcriptional level. Our results provide significant information for selecting and genetically improving pak choi cultivars with high nutritional quality.

## 2. Results

### 2.1. Metabolic Analysis of Pak Choi Leaves

**Figure 1:**
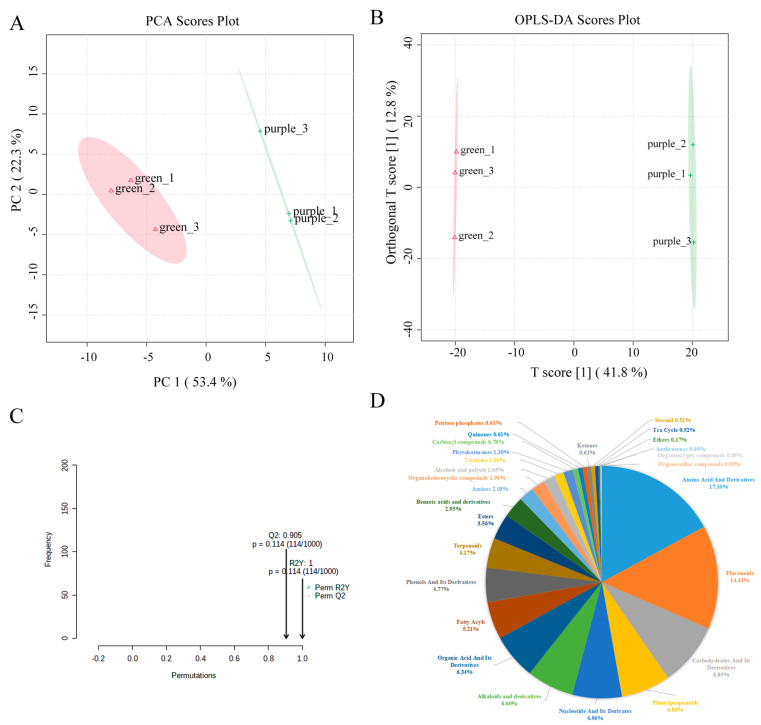
(**A**). Principal component analysis (PCA) score plots for pak chois. (**B**). Orthogonal partial least squares discriminant analysis (OPLS−DA) score plots for pak chois. (**C**). OPLS−DA permutation of the pak choi metabolic contents. (**D**). Categories and percentage of metabolites detected.

To compare the content of metabolites in green and purple pak choi, we analysed the leaf samples of the two varieties via LC-ESI-QTRAP-MS/MS. In total, 593 metabolites were identified in positive-ion mode and 559 metabolites were identified in negative-ion mode. The results of PCA indicated that there were large differences in the total number of metabolites between green and red pak choi, as well as the high similarity between biological replicates (Figure 1A). Furthermore, supervised orthogonal partial least squared discriminant analysis (OPLS-DA) was employed to predict the sample categories, and the results showed that the two groups of samples were significantly separated with a stable and reliable model (R2Y = 1 and Q2 = 0.905) (Figure 1B,C).

The 1152 identified metabolites can be classified into 27 categories, but most of them were classified into 7 categories: amino acids and their derivatives (17.10%), flavonoids (14.41%), carbohydrates and their derivatives (8.85%), phenylpropanoids (6.86%), nucleotides and their derivatives (6.86%), alkaloids and their derivatives (6.60%), and organic acids and their derivatives (6.34%) (Figure 1D and Table S1). Overall, the results showed that there were distinct metabolite profiles in green and purple pak choi leaves.

### 2.2. Metabolomic Difference between Green and Purple Pak Choi Leaves

**Figure 2:**
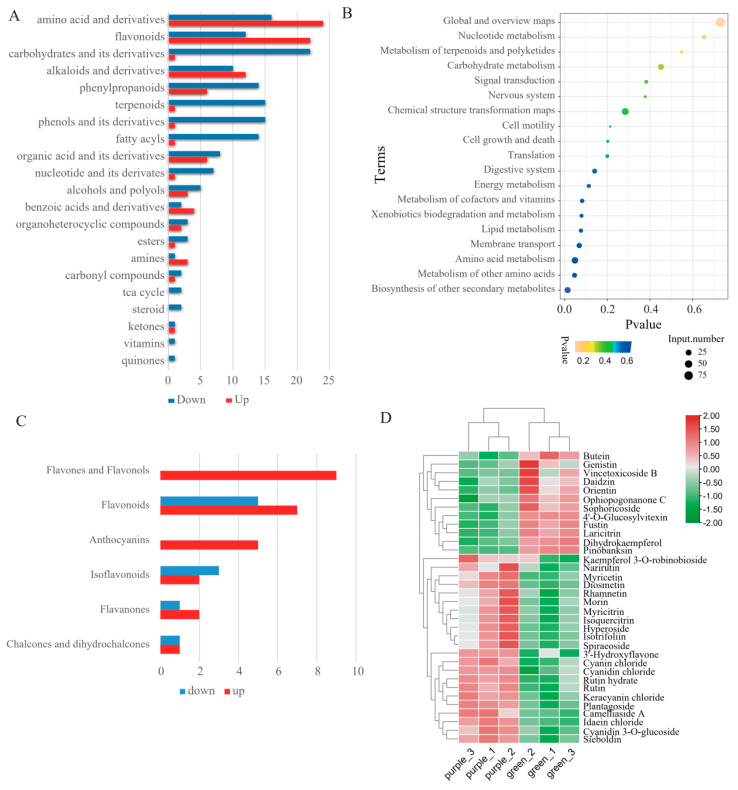
(**A**). The number of upregulated metabolites and downregulated metabolites in purple leaves relative to green ones for each category. (**B**). KEGG pathway enrichment analysis for DEMs. (**C**). The number of significantly up− or downregulated flavonoids. (**D**). Heatmap of 34 flavonoids that significantly differed between green and purple pak choi leaves.

A total of 246 DEMs were detected between green and purple pak choi leaves (Table S2). Of these DEMs, 90 were upregulated and 156 were downregulated in purple variety relative to the green variety. The DEMs were classified into 21 categories, and most of them were amino acids and their derivatives (16.26%), flavonoids (13.82%), carbohydrates and their derivatives (9.35%), alkaloids and their derivatives (8.94%), phenylpropanoids (8.13%), terpenoids (6.50%), phenols and their derivatives (6.50%), fatty acyls (6.10%), and organic acids and their derivatives (5.69%) (Table S3). Of these categories, amino acids and their derivatives, flavonoids, alkaloids, and their derivatives had more upregulated than downregulated metabolites in purple leaves relative to green ones. Carbohydrates and their derivatives, phenylpropanoids, terpenoids, phenols and their derivatives, and fatty acyls had more downregulated than upregulated metabolites in purple leaves than in green leaves (Figure 2A).

In addition, KEGG pathway enrichment analysis (*p* value < 0.05) showed that these DEMs were primarily significantly enriched in the biosynthesis of other secondary metabolites, the metabolism of other amino acids, and amino acid metabolism (Figure 2B), which were mainly flavonoids and amino acids and their derivatives. These pathways help to explain the metabolic processes underlying different leaf colours in pak choi.

### 2.3. Differential Accumulation of Flavonoids between Green and Purple Pak Choi Leaves

Flavonoids are one of the metabolites that have significant differences between purple and green pak choi leaves and are the main metabolites that affect the formation of purple leaves. In total, 166 flavonoids were identified, which can be classified into 35 flavones and flavonols, 17 anthocyanins, 16 flavanones, 5 isoflavonoids, 3 catechin derivatives, 4 chalcones and dihydrochalcones, 1 xanthone, and 85 other flavonoids (Table S4). Among these flavonoids, 34 flavonoids significantly differed between green and purple pak choi leaves (Table S5). In total, 24 flavonoids were upregulated, while the other 12 flavonoids were downregulated in purple pak choi (Table S5). Among these DEMs, all nine flavones and flavonols and five anthocyanins were upregulated in purple pak choi leaves. Most compounds in flavonoids and flavanones were significantly higher in purple than in green pak choi (Figure 2C). Furthermore, isoflavonoids had more downregulated DEMs in purple pak choi. We deduced a heatmap to show the changes in 34 flavonoids in green and purple leaves (Figure 2D). The top five with the highest fold change in upregulated flavonoids were all anthocyanins, namely cyanin chloride, cyanidin chloride, keracyanin chloride, idaein chloride, and cyanidin 3-O-glucoside. These data indicated that increased anthocyanin contents, particularly the top five highest up-accumulated anthocyanins, can positively regulate purple colour formation in purple pak choi.

### 2.4. Transcriptome Profiles of Pak Choi Leaves

**Figure 3:**
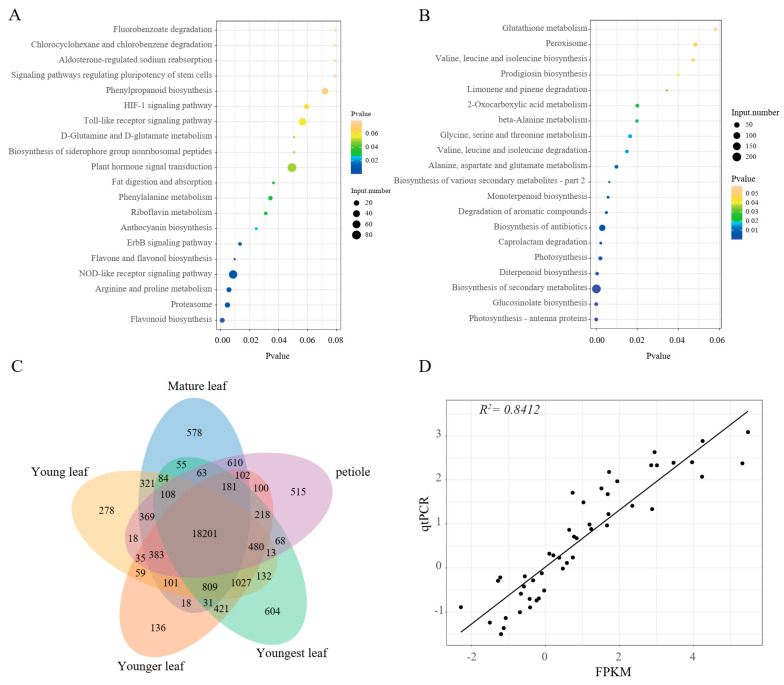
(**A**). Bubble charts showing the KEGG enrichment of upregulated DEGs. The *X*−axis of each bubble chart shows the metabolite number. The greater the bubble size, the greater the number of metabolites involved. The colour of bubble changes from purple to blue to green to yellow, which represents the *p* values of the enrichment analysis. (**B**). Bubble charts showing the KEGG enrichment of downregulated DEGs. (**C**). Venn diagram of gene expression characteristics of purple pak choi in 5 tissues. (**D**). Correlation analysis of expression changes detected by qPCR (*Y*−axis) and RNA-seq (*X*−axis).

To investigate the activation of the anthocyanin biosynthesis pathway in purple pak choi leaves, the RNA-seq of the purple and green leaf samples was conducted to quantify gene expression changes. Differentially expressed genes (DEGs) between the purple and green samples were determined. In total, 3897 upregulated and 3120 downregulated transcripts were identified. KEGG enrichment indicated that the upregulated DEGs were primarily related to flavonoid biosynthesis, flavone and flavonol biosynthesis, anthocyanin biosynthesis, as well as the proteasome and the NOD-like receptor signalling pathway (Figure 3A). The downregulated DEGs were abundant in photosynthesis–antenna proteins, glucosinolate biosynthesis, the biosynthesis of secondary metabolites, diterpenoid biosynthesis, photosynthesis, and so on (Figure 3B). In addition, the gene expression characteristics of purple pak choi in different tissues were further analysed using a Venn diagram (Figure 3C). The number of genes expressed in all tissues was 18,201, accounting for 69.69% of the total number of genes expressed.

To verify the credibility of the transcriptome data, we analysed the expression patterns of 12 flavonoid structural genes using qRT—PCR in all samples. The correlation coefficient (R2 = 0.8412) value between qPCR and fragments per kilobase of transcript per million mapped reads (FPKM) was high, implying that the RNA-seq data are valid and reliable.

### 2.5. Differential Expression of Flavonoid Structural Genes

**Figure 4:**
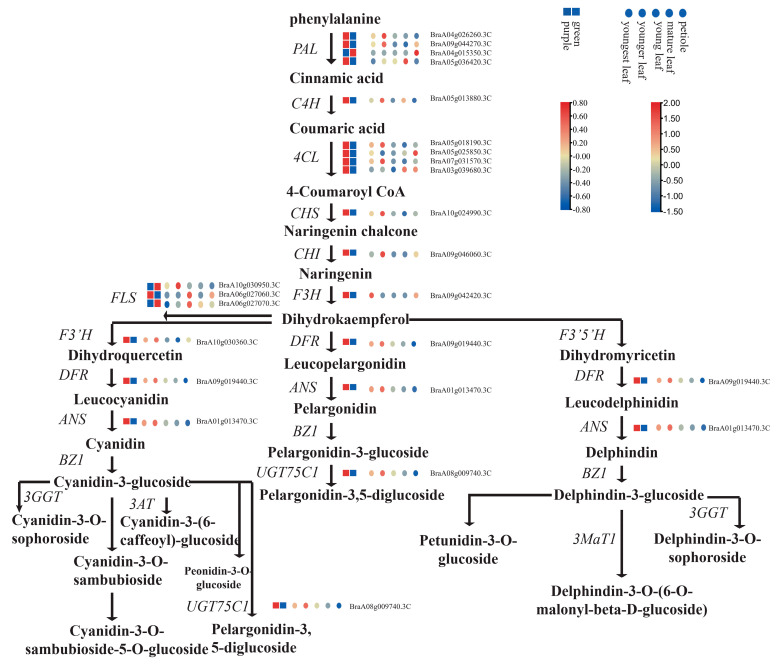
Transcript profiling of genes in the flavonoid biosynthetic pathway in purple and green pak choi. The square represents the relative expression of flavonoid pathway genes in purple and green pak choi, and the circle represents the expression of five leaf organs of purple pak choi. The red and blue colours indicate higher and lower gene expression abundances, respectively.

To investigate the accumulation of flavonoids in pak choi leaves in more detail, the gene expression patterns in flavonoid pathways were examined. Hence, genes that encode proteins related to anthocyanin biosynthesis were identified. In total, 19 pak choi genes were found to be involved in the biosynthesis of flavonoids, most of which (16 of 19) had higher expression in purple pak choi than in green pak choi. Thirteen genes displayed significantly different expression levels between the purple and green varieties (Table S6). Upregulated DEGs in purple pak choi were annotated as *PAL* (*BraA04g026260.3C*, *BraA05g036420.3C*), *4CL* (*BraA07g031570.3C*), *CHS* (*BraA10g024990.3C*), *CHI* (*BraA09g046060.3C*), *F3H* (*BraA09g042420.3C*), *F3’H* (*BraA10g030360.3C*), *FLS* (*BraA10g030950.3C*, *BraA06g027060.3C*, *BraA06g027070.3C*), *DFR* (*BraA09g019440.3C*), *ANS* (*BraA01g013470.3C*), *UGT79B1 (BraA06g021610.3C*), *UGT75C1* (*BraA08g009740.3C*), *and UGT78D2 (BraA02g006790.3C)* (Figure 4). All structural genes in the anthocyanin metabolism pathway had at least one copy and higher expression levels in purple pak choi than in green pak choi. In particular, *CHS*, *DFR*, *ANS*, and *UGT75C1* were much more highly expressed in purple pak choi than in green pak choi. In addition, the expression of one copy of *PAL* (*BraA04g015350.3C*) and two copies of *FLS* (*BraA10g030950.3C* and *BraA06g027070.3C*) in green leaves was higher than that in purple leaves, indicating that the two genes may not play a role in anthocyanin synthesis in leaves.

The expression changes in flavonoid biosynthesis genes in five organs in purple pak choi were also identified (Figure 4). Most copies of anthocyanin genes, including two copies of *PAL* and *C4H*; two copies of *4CL*, *CHS*, *CHI*, *DFR*, and *ANS*; and one copy of *FLS*, *F3′H*, and *UGT75C1*, were most highly expressed in younger leaves, which were just beginning to appear purple (Table S7). The results indicated that the highest expression of the anthocyanin gene did not occur at the time of the highest content of anthocyanin in leaves, but instead at the time of initial anthocyanin synthesis.

### 2.6. Analysis of Key Transcription Factors for Regulating Flavonoid Synthesis

**Figure 5:**
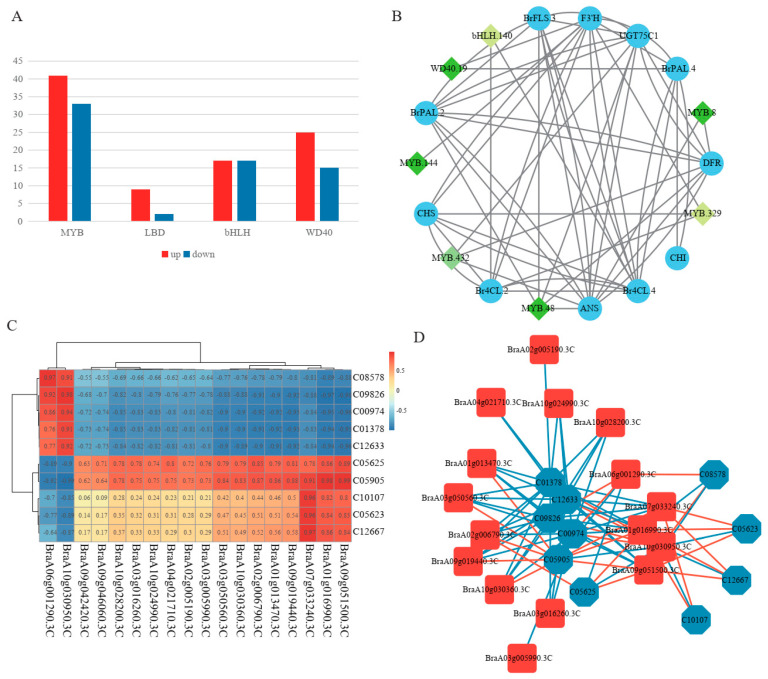
(**A**). The number of upregulated and downregulated genes in 4 different types of transcription factor gene families (MYB, bHLH, WD40, and LBD). (**B**). Correlation analysis of the associations between 4 transcription factor gene families related to flavonoid and anthocyanin biosynthesis genes. Green diamonds represent transcription factors and blue circles represent structural genes. (**C**). Correlation heatmap of DEGs and DEMs involved in flavone and flavonol biosynthesis (ko00944) and the flavonoid biosynthesis pathway (ko00941). (**D**). The correlation network of DEGs and DEMs involved in flavone and flavonol biosynthesis and the flavonoid biosynthesis pathway with a *p* value less than 0.05 and an absolute correlation coefficient value greater than 0.8. The blue hexagon represents the metabolites, the red square represents the genes, the red line represents the positive correlations, and the blue line represents the negative correlations.

Anthocyanin biosynthesis is regulated by TFs, including MYB, bHLH, WD40, and LBD, in plants. In this study, a total of 997 TFs were annotated to the four TF gene families (Table S8). Among them, 159 TFs differentially expressed between purple and green pak choi leaves were examined, including 74 MYBs (41 upregulated and 33 downregulated), 11 LBDs (9 upregulated and 2 downregulated), 34 bHLHs (17 upregulated and 17 downregulated), and 40 WD40 (25 upregulated and 15 downregulated) (Figure 5A and Table S9). The upregulated TFs may contribute to anthocyanin accumulation in purple pak choi, but further research is needed.

To identify the key TFs among these TFs, correlation analysis was conducted between the four different types of TFs (MYB, bHLH, WD40, and LBD) related to flavonoid and anthocyanin biosynthesis genes using the expression data of five organs in purple pak choi. Filtering was performed based on a correlation coefficient greater than 0.9 and a *p* value lower than 0.05. The results showed that the expression of 11 anthocyanin biosynthesis genes (*BrPLA.2*, *BrPLA.4*, *Br4CL.2*, *Br4CL.4*, *CHS*, *CHI*, *ANS*, *F3′H*, *BrFLS.3*, *DFR*, and *UGT75C1*) was strongly correlated (Figure 5B). Moreover, the correlation analysis of TFs showed that five *MYB*s (*MYB.8*, *MYB.48*, *MYB.144*, *MYB.329*, and *MYB.432*), one *bHLH* (*bHLH.140*), and one *WD40* (*WD40.19*) were strongly correlated with these anthocyanin biosynthesis genes. These results indicated that these 7 TFs may regulate the expression of 11 anthocyanin genes.

### 2.7. Association Analysis of the Transcriptome and Metabolome

The DEGs and DEMs involved in flavone and flavonol biosynthesis (ko00944) and the flavonoid biosynthesis pathway (ko00941) are shown in the correlation heatmap (Figure 5C). Five flavonoid DEMs, including C05625 (rutin), C05905 (cyanidin chloride), C10107 (myricetin), C05623 (isoquercitrin), and C12667 (quercetin 3-O-sophoroside), were positively correlated with most of the related flavonoid DEGs (16 of 18). Of these five DEMs, cyanidin chloride had the largest number of genes with correlation coefficients greater than 0.8. Three genes, *BraA07g033240.3C*, *BraA01g016990.3C*, and *BraA09g051500.3C*, which are copies of *CCoAOMT* (*caffeoyl-CoA O-methyltransferase*), are positively correlated with the DEMs with high correlation. The other five flavonoid DEMs, namely C08578 (butein), C09826 (pinobanksin), C00974 (dihydrokaempferol), C01378 (fustin), and C12633 (laricitrin), were negatively correlated with most of the related flavonoid DEGs (Figure 5D).

## 3. Discussion

Purple varieties of pak choi have been included in breeding due to their high anthocyanin content, which is associated with various health benefits. These varieties are also utilized in the production of natural colouring agents and functional foods. Hence, the purple colour serves as a quality criterion in pak choi breeding. In recent years, the composition and biosynthesis of anthocyanins in brassica vegetables, such as heading Chinese cabbage (*Brassica rapa* L. ssp. *pekinensis*) [21], mizuna (*Brassica rapa* L. var. *japonica*) [22], purple cai-tai (*Brassica rapa* L. var. *purpurea*) [23], ornamental cabbage or kale (*Brassica oleracea* var. *acephala*) [24,25], mustard (*Brassica juncea*) [26], and radish (*Raphanus sativus* L.) [27], have attracted extensive attention. The present study aimed to compare the metabolic and transcriptomic profiles between purple and green pak choi (*Brassica rapa* var. *chinensis*), as well as investigate the underlying mechanisms that contribute to differences in their colour and nutritional value.

Our results indicated that the metabolite profiles of pak choi leaves were abundant in amino acids and derivatives and flavonoids, which is similar to the results in a previous report [4]. In addition, there is a significant difference in the metabolite biosynthesis profiles between the two types of pak choi. Amino acids and their derivatives, flavonoids, alkaloids and their derivatives, which are all secondary metabolites that serve as antioxidants, were higher in purple leaves. Specifically, the purple pak choi exhibited significantly higher levels of anthocyanins, which are pigments responsible for the purple colour. The top five with the highest fold change in up-accumulated flavonoids are all anthocyanins, including cyanin chloride, cyanidin chloride, keracyanin chloride, idaein chloride, and cyanidin 3-O-glucoside, indicating that cyanidin and its derivatives are the main components affecting pak choi colour, which is similar to red kale results [25]. On the other hand, green pak choi had higher levels of carbohydrates and their derivatives because green leaves have more chloroplasts that produce more organic material during photosynthesis.

The differences in metabolite profiles between the two pak choi varieties were mainly due to differences in the genetic makeup. The increased accumulation of anthocyanins in plants is often attributed to the overexpression of genes related to late anthocyanin biosynthesis and positive TFs [28]. For example, pink-leaved ornamental kale showed higher levels of anthocyanin accumulation as a result of the ectopic expression of *BoDFR1* [29]. To further explore the molecular mechanisms regulating these differences in metabolite biosynthesis, we performed transcriptomic analyses and identified DEGs involved in various metabolic pathways. Notably, most DEGs related to flavonoid biosynthesis were upregulated in the purple pak choi leaves, which suggests that anthocyanin accumulation is caused by the upregulated expression of all structural genes. In addition, the key enzymes (*CHS*, *DFR*, *ANS* and *UGT75C1*) were much more highly expressed in purple pak choi than in green pak choi. This finding aligns with the conclusion drawn by Park et al. in their study on Mizuna [22]. Therefore, we considered that the increased expression levels of *CHS* resulted in increased dihydrokaempferol, which was further converted into dihydroquercetin. Then, the high expression of *DFR*, *ANS*, and *UGT75C1* produced much more cyanidin and its derivatives, leading to purple colour formation in pak choi (Figure 4).

TFs regulating anthocyanin structural genes play a key role in anthocyanin accumulation in Brassica vegetable tissues. In this study, we constructed regulatory network maps of TFs and flavonoid structural genes and explored their regulatory co-expression relationships (MYB, bHLH, WD40, and LBD). We found that 11 genes in the flavonoid or anthocyanin biosynthesis pathway were regulated by five MYB sites, one bHLH site, and one WD40 TF site. These results indicated that there are correlations between these TFs, which may have related regulatory functions. Among the seven genes, only *BraA07g035710.3C* (*MYB.329*), which is a homologous gene of *MYB2*, was significantly expressed in purple leaf pak choi (FDR < 0.01) at a much higher rate than in green leaf pak choi. Recent investigations have demonstrated that *MYB2* positively regulates the purple trait in purple cauliflower (*Brassica oleracea* L. var. *botrytis*) [30,31] and purple head Chinese cabbage (*Brassica rapa* L.) [32], but negatively controls anthocyanin accumulation in red cabbage (*B. oleracea* L. var. *capitata*) [33] and zicaitai (*Brassica rapa* L. ssp. *chinensis* var. *purpurea*) [34]. Therefore, the *MYB2* gene in purple pak choi has the opposite function to that of zicaitai and red cabbage. However, it does exhibit a positive regulatory function in anthocyanin accumulation, similar to cauliflower and purple head Chinese cabbage.

*CCoAOMT* is an important enzyme for lignin biosynthesis in plants [35]. Previous studies have shown that the downregulation of *PhCCoAOMT* in *Petunia hydrida* can activate anthocyanin accumulation [36], but Xi et al. indicated that *CCoAOMT* was upregulated in purple caitai (*Brassica compestris*. var. *tsai-tai* Hort.). In our study, *CCoAOMT* was upregulated in purple pak choi and positively correlated with flavonoid-related DEMs, which is consistent with the results on caitai. The dual role of *CCoAOMT* in anthocyanin biosynthesis is mainly due to species differences.

## 4. Materials and Methods

### 4.1. Plant Material and Sampling

The pak choi varieties ‘524′ (green) and ‘B1204′ (purple) were chosen as the research materials. The seeds were cultivated in a 50-well tray and grown in a glass greenhouse at the Qiaosi experimental farm of the Zhejiang Academy of Agricultural Sciences in September 2021. After 15 days, the seedlings were transplanted to the open field. Pak choi leaf samples were harvested 60 d after transplantation in November 2021. Each sample had three biological replicates and each repeat included five individual plants. The sampled pak choi leaves were wrapped in tin foil, frozen in liquid nitrogen, and stored at −80 °C until use. Leaves that fully developed at harvest time were used for metabolic analysis and the transcriptomic analysis of purple and green leaf differences. Four leaves that were separated from plant materials from outer rosette leaves to the inner, named sequentially as mature, young, younger, and youngest leaves, as well as petiole, were also used for transcriptomic analysis.

### 4.2. Sample Preparation and Metabolite Extraction

Each leaf sample (100 mg) was ground with liquid nitrogen, and the homogenate was suspended in 500 μL of prechilled 80% methanol containing 0.1% formic acid via thorough vortexing. The samples were incubated on ice for 5 min and then centrifuged at 15,000× *g* and 4 °C for 20 min. The supernatant was diluted with LC—MS-grade water (Thermo Fisher Scientific, Waltham, MA, USA) to a final concentration of 53% methanol. The samples were then transferred to fresh Eppendorf tubes and centrifuged at 15,000× *g* for 20 min at 4 °C. Finally, the supernatant was used for further LC–MS/MS (Thermo Fisher Scientific, Waltham, MA, USA) analysis. An equal amount of extract from each sample was mixed and used as a quality control (QC) sample [37].

### 4.3. LC–MS/MS Analysis

The ExionLC™ AD system and QTRAP^®^ 6500 + mass spectrometer equipped with electrospray ionization (ESI) sources operating in positive and negative ion modes (AB SCIEX, Framingham, MA, USA) were used for LC—MS/MS analysis. Samples were injected into an Xselect HSS T3 column (2.5 μm, 2.1 × 150 mm) at a flow rate of 0.4 mL/min at 40 °C. The mobile phase consisted of 0.1% formic acid–water (phase A) and 0.1% formic acid–acetonitrile (phase B), and the solvent gradient was set as follows: 2% B, 0 min; 2% B, 2 min; 100% B, 15 min; 100% B, 17 min; 2% B, 17.1 min; and 2% B, 20 min. The MS source conditions were set as follows: curtain gas: 35 psi; collision gas: medium; ion spray voltage: 5500 V; temperature: 550 °C; ion source gas 1: 60; and ion source gas 2: 60. The negative polarity mode operated with the same parameters as the positive mode, except for an ion spray voltage of −4500 V.

### 4.4. Identification of Metabolites

Metabolite identification was performed based on the Novogene database (novoDB) (Tianjing, China) in multi-reaction monitoring mode [38]. Compounds were quantified according to Q3 (sub-ions) and qualitatively analysed by Q1 (parent ions), Q3 (sub-ions), RT (retention time), DP (declustering voltage), and Ce (collision energy). SCIEX OSV1.4 software was used to open the off-machine file for the integration and correction of chromatographic peaks.

The identified metabolites were annotated according to public MS databases, including the KEGG compound database [39], the HMDB database [40], and the LIPIDMaps database [41]. Moreover, the identified metabolites were subjected to principal component analysis (PCA) and orthogonal partial least squares discriminant analysis (OPLS-DA) with MetaWare Cloud, a free online platform used for data analysis (https://cloud.metware.cn (accessed on 12 March 2023)), using log2-transformed data. Metabolites with VIP > 1, *p* values < 0.05, and fold changes ≥ 2 or ≤ 0.5 were considered to be differential metabolites (DEMs).

### 4.5. Transcriptome Sequencing and Differentially Expressed Gene Analysis

The total RNA of samples was extracted using TRIzol^®^ Reagent according to the manufacturer’s instructions (Invitrogen, Carlsbad, CA, USA). Then, RNA quality was determined using a 2100 Bioanalyzer (Agilent) and quantified using an ND-2000 (NanoDrop, Wilmington, DE, USA). High-quality RNA (OD260/280 = 1.8~2.2, OD260/230 ≥ 2.0, RIN ≥ 6.5, 28S:18S ≥ 1.0, > 10 μg) was sent to Biozeron Technologies Co., Ltd. (Shanghai, China) for library construction and sequencing. Each biological replicate established a transcriptome library, following the TruSeqTM RNA Sample Preparation Kit instructions from Illumina (San Diego, CA, USA), and were sequenced using a NovaSeq 6000 sequencer (Illumina, San Diego, CA, USA).

Low-quality reads and reads containing adapters and poly-N were removed from the raw data. Then, the clean data were mapped to the *Brassica rapa* genome (Chiifu V3.0 genome) [42] using Hisat2 software [43]. The expression abundance of reads was represented by the fragments per kilobase million (FPKM) value. The differentially expressed genes (DEGs) were selected with a threshold of |log2 Ratio| ≥ 1 and a FDR ≤ 0.05. The transcripts were annotated from public databases, including the NCBI nonredundant protein (Nr) database, the Kyoto Encyclopedia of Genes and Genomes (KEGG) database, and the Gene Ontology (GO) database. GO functional enrichment and KEGG pathway analysis were performed using Goatools (https://github.com/tanghaibao/Goatools, accessed on 6 October 2022) and KOBAS (http://kobas.cbi.pku.edu.cn/home.do, accessed on 6 October 2022). DEGs were significantly enriched in GO terms and metabolic pathways when their Bonferroni-corrected *p* value was less than 0.05.

### 4.6. Coexpression Network Analysis for the Construction of Modules

DEGs and DEMs were obtained from transcriptome and metabolome data, respectively, through comparisons between two green and purple pak choi, each with three biological replicates. KEGG enrichment analysis was conducted separately for each piece of omics data, and DEGs and DEMs enriched in the same KEGG database were analysed for association analysis. Correlations between gene expression and metabolite abundance were measured using Pearson correlation coefficients with values ranging from −1 to +1. The association between the metabolome and transcriptome data was evaluated using R language software (https://cran.r-project.org/bin/windows/base/, accessed on 11 July 2022), with a *p* value threshold less than 0.05 and an absolute correlation coefficient value greater than 0.8. Heatmaps and network maps were visualized using the pheatmap package [44] and Cytoscape [45], respectively.

### 4.7. Gene Validation via Real-Time Quantitative PCR (qRT—PCR)

The qRT—PCR verification of selected DEGs was conducted on the high-quality RNA samples used for RNA sequencing above. First-strand cDNA was synthesized using PrimeScript^®^ Reverse Transcriptase (Takara Biotechnology, Dalian, China). PCR was carried out on a Stratagene Mx3000P thermocycler (Agilent, Santa Clara, CA, USA) using a SYBR Green qPCR Kit (TaKaRa Biotechnology, Dalian, China) following the manufacturer’s protocol under the following program: 95 °C for 5 min, followed by 40 cycles of 95 °C for 15 s and 60 °C for 30 s. Actin was utilized as an internal control [46], and the relative gene expression levels were calculated with the 2^−ΔΔCt^ method. Gene-specific primers were designed using the Primer Premier 3.0 program (Table S10). qRT—PCR was performed and included three biological replicates, each with three technical replicates. The correlation ratio between RNA-seq and qRT—PCR in each tissue and time point was calculated and drawn by R programming language with changed log2 values.

## 5. Conclusions

Overall, our study provides new insights into the metabolic and transcriptomic differences between purple and green pak choi and highlights the potential mechanisms that underly these differences. In total, 246 DEMs were detected between green and purple pak choi leaves, which are mainly amino acids and their derivatives and flavonoids. Thirty-four flavonoids significantly differed between green and purple pak choi leaves. Further analysis showed that cyanidin and its derivative anthocyanins were abundant in purple pak choi. The different expression of DEGs related to flavonoid biosynthesis pathways, especially *CHS*, *DFR*, *ANS*, and *UGT75C1*, might cause the different accumulation levels of anthocyanin derivatives. *MYB2* is the key TF that plays a positive regulatory role in anthocyanin accumulation in purple pak choi. These findings may have important implications for the development of new varieties of pak choi with enhanced nutritional quality and aesthetic appeal.

## Data Availability

We have deposited our raw data in the China National GeneBank DataBase (CNGBdb) under accession numbers CNP0004208 (https://db.cngb.org/search/project/CNP0004208/).

## Supplementary Materials

The following supporting information can be downloaded at: https://www.mdpi.com/article/10.3390/ijms241813781/s1.
